# Treatment preferences in juvenile idiopathic arthritis – a comparative analysis in two health care systems

**DOI:** 10.1186/1546-0096-11-3

**Published:** 2013-01-15

**Authors:** Boris Hugle, Johannes-Peter Haas, Susanne M Benseler

**Affiliations:** 1 Division of Rheumatology, Department of Paediatrics, The Hospital for Sick Children, University of Toronto, 555 University Avenue, Toronto, ON, M5G 1X8, Canada; 2 German Center for Pediatric Rheumatology, Garmisch-Partenkirchen, Germany; 3 Child Health Evaluative Sciences, Research Institute, The Hospital for Sick Children, University of Toronto, Toronto, Canada

**Keywords:** Juvenile idiopathic arthritis, Treatment preferences, Survey, Austria, Canada, Germany, Physiotherapy, Funding

## Abstract

**Background:**

Variations in the treatment of juvenile idiopathic arthritis (JIA) may impact on quality of care. The objective of this study was to identify and compare treatment approaches for JIA in two health care systems.

**Methods:**

Paediatric rheumatologists in Canada (n=58) and Germany/Austria (n=172) were surveyed by email, using case-based vignettes for oligoarticular and seronegative polyarticular JIA. Data were analysed using descriptive statistics; responses were compared using univariate analysis.

**Results:**

Total response rate was 63%. Physicians were comparable by age, level of training and duration of practice, with more Canadians based in academic centres. For initial treatment of oligoarthritis, only approximately half of physicians in both groups used intra-articular steroids. German physicians were more likely to institute DMARD treatment in oligoarthritis refractory to NSAID (p<0.001). Canadian physicians were more likely to switch to a different DMARD rather than a biologic agent in polyarthritis refractory to initial DMARD therapy. For oligoarthritis and polyarthritis, respectively, 86% and 90% of German physicians preferred regular physiotherapy over home exercise, compared to 14% and 15% in Canada. Except for a Canadian preference for naproxen in oligoarthritis, no significant differences were found for NSAID, intra-articular steroid preparations, initial DMARD and initial biologic treatment.

**Conclusions:**

Treatment of oligo- and polyarticular JIA with DMARD is mostly uniform, with availability and funding obviously influencing physician choice. Usage of intra-articular steroids is variable within physician groups. Physiotherapy has a fundamentally different role in the two health care systems.

## Background

Juvenile idiopathic arthritis (JIA) is the most common rheumatic disease in childhood. Children with JIA suffer from chronic pain and frequently experience considerable limitations in their daily life [1]. Current therapy concepts concentrate on early aggressive treatment to prevent long-term damage. The International League of Associations for Rheumatology classification divides JIA into several subtypes and has allowed a rational approach to subgroup-specific treatment [2].

JIA is mainly treated with a combination of anti-inflammatory and immunomodulatory agents, in combination with physical and occupational therapy [3]. Introduction of disease-modifying anti-rheumatic drugs (DMARD) and, more recently, biologic agents such as TNF-antagonists, have significantly changed the treatment over the last two decades [4]. Various professional societies and groups have put considerable effort into developing recommendations and guidelines for the treatment of JIA [5,6].

Substantial variability in treating rheumatic disease has been described previously on practically all aspects of patient care [7]. Studies examining treatment preferences within countries have shown differences even in straightforward procedures, such as joint injections [8]. Treatment choices are influenced not only by current evidence, but also by established practice, individual experience and, last but not least, availability of certain drugs and treatment modalities [9,10].

Previous surveys on treatment preferences in JIA have concentrated on single countries or comparable health care systems [11,12]. Surveys of treatment modalities for various subtypes of JIA have been performed by professional organisations within one country with the focus of establishing consensus and formulating guidelines [5,13]. Health care systems in different countries put varying emphasis on certain aspects of patient care. A comparison of treatment approaches between health care systems offers opportunities to improve health care by pointing out differences in disease concepts and therapeutic approaches.

This is the first comparison between health care systems of treatment preferences in JIA to date. The aim of this study was to identify patterns of treatment preferences for JIA using a case-based evaluation of paediatric rheumatologists in two different health care systems, Germany/Austria and Canada.

## Methods

### Participants and survey modalities

A 20-item self-administered multiple-choice questionnaire (see Additional files 1 and 2) was developed for this study, using a web-based tool (SurveyMonkey.com LLC, Palo Alto, CA). The questionnaire was critically reviewed by the authors and 2 other paediatric rheumatologists and was modified following their input. It was translated into German by a native German speaker (BH).

For Germany and Austria, a member list from the Gesellschaft für Kinder- und Jugendrheumatologie (Society for Paediatric and Adolescent Rheumatology, GKJR) was obtained. The GKJR represents the subspecialty of paediatric rheumatology in both countries, Germany and Austria. As there is no comparable representative body in Canada, a member list of the Canadian Alliance for Paediatric Rheumatology Investigators (CAPRI) was obtained. Inactive members or members practicing outside their respective country were removed from the list.

An email inviting possible participants and containing the hyperlink to the questionnaire was mailed to 172 members of the GKJR and 58 members of the CAPRI. The email was sent again three times in weekly intervals to previous non-responders.

### Questionnaire design

The questionnaire addressed the following domains:

I. *Demographics:* Questions were asked about demographic data of the participants, including age, gender, years since graduation, level of training including board certification for paediatrics and paediatric rheumatology, practice setting (dividing into hospital-based, academic; hospital-based, non-academic; and community-based) and country of practice.

II. *Treatment approach to oligoarticular JIA:* Respondents were asked how they would treat a 3 year old girl with a typical presentation of oligoarticular JIA (ANA-positive) with knee and ankle joints affected. Case-specific questions were asked regarding (1) initial treatment of oligoarthritis, (2) treatment of refractory oligoarthritis, (3) treatment of complications (uveitis refractory to topical steroids).

III. * Treatment approach to polyarticular JIA:* Respondents were asked how they would treat a 14 year old girl with a typical presentation of seronegative polyarticular JIA (ANA-positive) with a total active joint count of 9 joints. Case-specific questions were asked regarding (1) initial treatment of seronegative polyarthritis, (2) treatment choice in seronegative polyarthritis refractory to DMARD, (3) time to treatment change in seronegative polyarticular JIA refractory to DMARD, and (4) time to institute biologic agents in seronegative polyarticular JIA refractory to DMARD.

IV. * Approach to physiotherapy in oligo- and polyarticular JIA.* In each of the two scenarios described under II) and III), a case-based question was asked regarding the approach to physiotherapy. Choices offered were: regular weekly physiotherapy by a trained physiotherapist; home exercise after initial coaching; or, encouragement of physical activity at home.

V. *Medication preferences in oligo- and polyarticular JIA.* Questions were asked regarding specific medication choices, including: initial choice of non-steroidal anti-inflammatory drugs (NSAID) preparation in a 3 year old girl with oligoarticular JIA; initial choice of NSAID preparation, DMARD preparation and biologic agent in a 13 year old girl with polyarticular JIA; and preferred corticosteroid for joint injection of the knee in JIA.

### Analysis

Baseline demographic data and treatment data were calculated using descriptive statistics. As both Germany and Austria operate on a very similar system of state-controlled mandatory health care, responders from both countries were considered as one group for purposes of statistical analysis [14]. For subgroup analysis, residents and paediatricians were also grouped together (compared to subspecialists). Univariate analysis was performed using Chi-squared analysis, Fischer’s exact test or Wilcoxon rank-sum test, where appropriate. Tests were performed at a 0.05 significance level except for subgroup comparison; here significance levels were adjusted for multiple comparisons as appropriate. Data are expressed as mean ± standard deviation (SD) unless stated otherwise. Statistical analysis was performed with SPSS version 17.0 (SPSS Inc., Chicago, USA).

## Results

Out of 230 paediatric rheumatologists polled, a total of 145 physicians (63.0%) participated in this study and completed the survey. Among 172 members of the GKJR, 108 (62.8%) responded. Of these, 100 practiced in Germany and 8 in Austria. Of 58 members of CAPRI, 37 (63.7%) participated.

### Demographics and baseline characteristics

Participating German/Austrian and Canadian paediatric rheumatologists were found to be comparable in age, gender and years since graduation from medical school. Respondents from Germany/Austria were less likely to have attained subspecialty board certification for paediatric rheumatology (70.6% vs. 88.2%; *P=*0.002) and were also significantly less likely to practise in an academic setting (39.1% vs. 97.1%; *P* < 0.001). Baseline characteristics are summarised in Table 1.

**Table 1:** Demographic characteristics of participating pediatric rheumatologists in Canada and Germany/Austria

	**German/Austrian physicians (N=109)**	**Canadian physicians**	P*
		**(N=38)**	
**Gender (% female)**	43.9%	67.0%	Ns
***Level of training***			
Pediatr. Rheumatologist**	70.6%	85.7%	
**Pediatrician, board-cert.**	24.8%	5.9%	0.002
**Resident**	4.6%	5.9%	
**Median age (range)**	45 years (30 – 70)	43 years (28 – 74)	Ns
Years since graduation***	19.6 ± 9.5 years	19.3 ± 12.0 years	Ns
***Type of practice***			
**Academic hospital**	39.1%	97.1%	
**Non-academic hospital**	50.5%	2.9%	<0.001
**Community-based**	10.5%	0%	
**Country**	Germany 91.4%	Canada 100%	
	Austria 8.6%		

*‘ns’ denotes *P>0.05.*

** board certification in paediatrics and subspecialisation in pediatric rheumatology.

*** Data are expressed as mean ± standard deviation.

### Treatment approach to oligoarticular JIA

#### Initial treatment

NSAID were suggested for initial treatment of JIA by 92.5% of German/Austrian and 86.1% of Canadian physicians. Intra-articular steroids were preferred by 56.1% of German/Austrian, and 47.2% of Canadian physicians. DMARDs were preferred by 7.5% and 11.1%, respectively. Further results are shown in Table 2.

**Table 2:** Comparison of preferred treatment approaches in JIA between Canadian and German/Austrian pediatric rheumatology practitioners

	**Germany/Austria (N=109)**	**Canada (N=37)**	***P*‡**
Initial treatment of oligoarticular JIA*			
**NSAID**	92.5	86.1	ns
**Intra-articular steroids**	56.1	47.2	ns
**DMARDs**	7.5	11.1	ns
**Oral steroids**	1.9	0	ns
**Biologic agents**	0.9	0	ns
Treatment of refractory oligoarticular JIA*			
**NSAID**	25.0	13.5	ns
**Intra-articular steroids**	96.2	100.0	ns
**DMARDs**	29.8	2.7	<0.001
**Oral steroids**	0	0	ns
**Biologic agents**	0	0	ns
Treatment of uveitis in oligoarticular JIA*			
**NSAID**	5.8	18.2	ns
**DMARDs**	89.3	84.9	ns
**Oral steroids**	17.5	3.0	0.043
**Biologic agents**	13.6	0	0.022
Initial treatment of polyarticular JIA*			
**NSAID**	85.3	77.8	ns
**Intra-articular steroids**	29.4	11.1	0.041
**DMARDs**	90.2	75.0	0.045
**Oral steroids**	34.3	19.4	ns
**Biologic agents**	1.0	0	ns
Preferred treatment of refractory polyarticular JIA**			0.002
**Switch to different DMARD**	2.1%	20.6%	
**Additional DMARD**	11.7%	14.7%	
**Intra-articular Steroids**	17.0%	17.6%	
**Add or switch to biologic agents**	69.1%	47.1%	
**Median time to change treatment of refractory polyarticular JIA (range)†**	3 months (1 – 9)	3 months (1 – 9)	ns
**Median time to switch to biologic agents in refractory polyarticular JIA (range)†**	3 months (1 – 9)	4 months (1 – 12)	ns
Preferred physiotherapy recommendation for oligoarticular JIA**			<0.001
**Regular physiotherapy**	85.6%	13.9%	
**Home exercise after initial coaching**	14.4%	52.8%	
**Encourage physical activity**	1.0%	36.1%	
Preferred physiotherapy recommendation for polyarticular JIA**			<0.001
**Regular physiotherapy**	89.5%	14.7%	
**Home exercise after initial coaching**	9.5%	73.5%	
**Encourage physical activity**	1.0%	11.8%	

* Multiple answer question, values denote percentage of participants giving a positive answer.

** Single answer question.

† Participants were given a choice of full months (range 1 – 24 months).

‡ ‘ns’ denotes *P>0.05.*

### Treatment of refractory oligoarticular JIA

For oligoarticular JIA refractory to NSAID, 96.2% of German/Austrian and 100% of Canadian physician chose treatment intra-articular steroids. A significantly higher proportion of German/Austrian compared to Canadian physicians indicated treatment with DMARD (29.8% vs. 2.7%, *P* < 0.001). 25.0% of German/Austrian physicians compared to 13.5% of Canadian physicians would treat with NSAID at this time.

#### Treatment of chronic uveitis

For treatment of chronic uveitis in the model patient with ANA-positive oligoarticular JIA refractory to topical steroids, 89.3% of German/Austrian and 84.9% of Canadian physicians suggested treatment with DMARD. Significant differences were found in the number of physicians who would start oral steroids (German/Austrian 17.5% vs. Canadian 3.0%, *P*=0.043) or biologic agents (German/Austrian 13.6% vs. Canadian 0%, *P=*0.022) at this time.

### Treatment approach to polyarticular JIA

#### Initial treatment of polyarticular JIA

DMARDs were preferred by 90.2% of German/ Austrian physicians vs. 75.0% of Canadian physicians (*P=*0.045), and 29.4% of German/Austrian vs. 11.1% of Canadian physician would use intra-articular steroids in this situation (*P=*0.41). No significant difference was found in the use of NSAID (German/Austrian 85.3% vs. Canadian 77.8%), oral steroids (34.3% vs. 19.4%) and biologic agents (1.0% vs. 0%), or for the choice of initial NSAID in polyarticular JIA (German/Austrian 74.5%, Canadian 94.4%).

#### Treatment of refractory polyarticular JIA

Significantly more Canadian than German/Austrian physicians would switch to a different DMARD (20.6% vs. 2.1%) in polyarticular JIA refractory to a DMARD, while German/Austrian physicians were more likely to start a biologic agent (69.1% vs. 47.1%, *P=*0.002).

#### Time to change treatment/time to institute biologic agent

No significant differences were found between German/Austrian and Canadian physicians in time to change treatment after institution of DMARD (4.2 ± 1.5 months vs. 4.2 ± 1.5 months). Similarly, the time to switch to a biologic agent after start of DMARD treatment was not significantly different in German/Austrian and Canadian physicians (5.1 ± 1.9 months vs. 5.7 ± 2.5 months).

### Approach to physiotherapy in oligo- and polyarticular JIA

In oligoarticular JIA, German/Austrian physicians significantly (p<0.001) more frequently recommended regular physiotherapy compared to Canadian physicians (85.6% vs. 13.9%). Canadian physicians opted more frequently for home exercise after initial coaching (52.8% vs. 14.4% of German/Austrian physicians) or for encouraging physical activity alone (36.1% vs. 1.0% of German/Austrian physicians).

Similarly, in polyarticular JIA German/Austrian physicians routinely opted for regular physiotherapy (89.5% vs. 14.7% of Canadian physicians, p<0.001), while their Canadian counterparts more frequently chose home exercise (73.5% vs. 9.5% of German/Austrian physicians) or encouragement of physical activity (11.8% vs. 1.0% of German/Austrian physicians).

### Medication preferences in oligo- and polyarticular JIA

Medication preferences showed significant differences only in the preference for NSAIDs in the treatment for oligoarticular JIA: Naproxen was almost uniformly preferred for initial treatment of oligoarticular JIA by Canadian physicians (97.2%). Medication preferences varied more among German/Austrian physicians, with most opting for either naproxen (43.8%), ibuprofen (34.3%) or indomethacin (20.0%, *P<*0.001). Naproxen was also the preferred preparation for choice of initial NSAID in polyarticular JIA, (German/Austrian 74.5%, Canadian 94.4%).

For intra-articular corticosteroid injection into a knee joint, triamcinolone hexacetonide was almost exclusively the preparation of choice in both health care systems (German/Austrian 98.0%, Canadian 100%).

For the treatment of seronegative polyarticular JIA, medication preferences were almost unanimous for choice of initial DMARD (MTX, German/Austrian and Canadian 100%) and initial biologic agent (etanercept, German/Austrian 98.1%, Canadian 94.5%).

### Differences between subgroups by training level

Answers from respondents with subspecialty qualifications and those who were residents or paediatricians without subspecialty were compared between and within countries. No significant differences were detected for treatment preferences, time to switch medications or medication preferences.

## Discussion

This study is the first to compare treatment of JIA in two different health care systems. Both these systems have universal health care. However, treatment modalities are reimbursed to varying degrees in the two systems, with medications being part of the publicly funded system in Germany but mostly covered by private insurance in Canada. The oligoarticular and seronegative polyarticular subtypes represented in this survey make up more than 50% of JIA cases in Canada and Germany [15,16]. With close to two thirds of polled physicians answering, this survey constitutes a representative sample of the practicing physician population in paediatric rheumatology in their respective countries, and a large majority of respondents had both subspecialist training and significant experience in the field.

Working with vignettes is a sophisticated and valid method for measuring the quality of care and medical decision-making [17,18]. Geographic variations in health care depending on the system have been shown in various studies [19,20]. Compared to other findings, the approach to medical treatment of JIA observed in this study is rather uniform [8,10,21]. The largest variations were found in switching to additional DMARDs or biologics after failing the first DMARD in the treatment of seronegative polyarthritis (Table 2). Nonetheless, the majority of respondents followed published evidence and guidelines. This reflects the increasing numbers of international clinical trials with uniform standard of practice; also, standardisation of paediatric rheumatology training programs has shaped the approach to treatment. The efforts of the professional societies for paediatric rheumatology in their respective countries to standardise treatment approaches by guidelines and recommendations represent a continuation of this trend.

Drug availability may have played a major part in the observed differences in medication preferences between the two health care systems. German and Austrian paediatric rheumatologists were more willing to use DMARDs in refractory oligoarticular JIA, as well as initial treatment of polyarticular JIA. They were also more ready to switch to biologic agents after failure of a single DMARD in polyarticular JIA, and possibly at an earlier time. In Canada, funding agencies frequently demand the patient to fail more than one DMARD before approving treatment with biologic agents, despite lack of evidence for this approach [22]. By contrast, both Germany and Austria have a state-governed system of mandatory health insurance which covers all licenced medications including biological without prior approval from the insurance companies (off-label uses are, however, exempt from this). Given that etanercept and adalimumab were both licensed for treatment of refractory JIA in Germany at the time of the survey, while leflunomide (as an alternative DMARD) was not, the observed differences are understandable.

Use of intra-articular steroids was remarkable, because only half of the physicians in both health care systems opted to treat an initial presentation of JIA this way. Intra-articular joint injections have been demonstrated to be of significant benefit in oligoarticular JIA, where as many as 70% of patients show no reactivation of disease for at least one year [23,24]. The patient presented in the questionnaire was described as having four active joints (both knee and ankle joints), which could explain the reluctance of practitioners. However, practice variations even in JIA presenting as monoarthritis have been described previously and have been attributed to both views of effectiveness of intra-articular steroids and proficiency with the procedure [8].

A striking difference is presented in the role of physiotherapy in the treatment of JIA between the two health care systems (Table 1). German/Austrian physicians showed a clear preference for regular weekly physiotherapy in a controlled setting, while most Canadian physicians were content with recommending a home-exercise program for the patient. In an open study on 25 children with polyarticular JIA, an 8-week physical conditioning program led to significant improvement in joint symptoms [25]. At least moderate adherence to an exercise program was associated with better function and less pain due to arthritis in one study on 175 Canadian children with JIA [26]. Exercise training in a randomised controlled trial in 80 children with JIA resulted in self-reported improved function measured by CHAQ, but not other tests [27]. However, a recent Cochrane review showed no statistically significant improvement of functional ability, quality of life or aerobic capacity by exercise programs [28]. The small number of open studies on land- and water-based exercise have been reviewed, leading to the suggestion that participation in a physiotherapy program at least twice a week for at least 6 weeks may help to reduce disease symptoms [29]. However, no study has directly compared efficacy of exercise programs versus controlled physiotherapy in JIA. A strong emphasis is placed on physiotherapy in the German/Austrian health care system especially; the German guidelines strongly state that ‘without adequate physiotherapy […], the treatment of patients with JIA is impossible’ [6]. This represents a fundamental difference from the approach to physiotherapy in Canada. However, it is unclear if this is also influenced by availability of the treatment modality; in Germany and Austria, health insurance usually covers physiotherapy in children, while in Canada, services are frequently privately funded or provided by non-government organisations such as the Arthritis Society.

This survey was limited by unequal sample and population sizes, with the German/Austrian respondent group being approximately three times larger. No unifying body comparable to the GKJR could be readily surveyed in Canada. As virtually all paediatric rheumatologists in Canada were members of CAPRI at the time of the survey, this was chosen as a substitute. The higher percentage of Canadian respondents working in academic centres is therefore not surprising. Differences in level of training and practice setting could also reflect the fact that paediatric rheumatology as a subspecialty has been introduced in most provinces of Germany and Austria only in the last decade and is not represented in all academic centres, while it has been recognized as a subspecialty in Canada since 1997. Nonetheless, both physician groups were comparable for age and experience. By design this survey could not address the fact that many children with JIA are treated by paediatricians and adult rheumatologists with no subspecialty training. Germany and Austria were considered having a sufficiently similar health care system and paediatric rheumatology tradition to group them together for purposes of statistical analysis. However, there are some differences especially in availability of certain drugs that might have influenced some choices. Numbers of Austrian paediatric rheumatologists were still too small for subgroup analysis. Respondents were also possibly influenced in their treatment choices by the subsequent changes in the patient vignettes; the number of polled physicians was insufficient to present multiple versions of this survey. Particular care was taken to present patients in as neutral a manner as possible.

## Conclusions

Our study demonstrates that the majority of paediatric rheumatologists follow established guidelines for pharmacological treatment, with a broader variation for intra-articular steroids. Funding and drug availability by manufacturers certainly has a strong influence on the treatment choices of the practitioners. The role of physiotherapy demonstrates a fundamental difference in disease concepts between Europe and North America. Given these disparities, future research should focus on the impact of physiotherapy on long-term outcomes in JIA.

## Competing interests

The authors declare that they have no competing interests.

## Authors’ contributions

BH and SMB designed and carried out the survey and the statistical analysis. JPH participated in the design and coordination of the study and helped draft the manuscript. All authors read and approved the final manuscript.

## Supplementary Material

Additional file 1 Text of electronic survey questionnaire from SurveyMonkey, english. Click here for file

Additional file 2 Text of electronic survey questionnaire from SurveyMonkey, german. Click here for file
