# Supplementary material for: Treatment preferences in juvenile idiopathic arthritis – a comparative analysis in two health care systems
Source: Pediatr Rheumatol Online J. 2013 Jan 15;11:3. doi: 10.1186/1546-0096-11-3 (PMC3573942; doi:10.1186/1546-0096-11-3)
Supplement: Additional file 1 — Text of electronic survey questionnaire from SurveyMonkey, english. [file 1546-0096-11-3-S1.pdf]

Bevorzugte Behandlungsmethoden bei JIA in Deutschland und Kanada

Sehr geehrte Frau Kollegin, sehr geehrter Herr Kollege,

Vielen Dank für Ihre Bereitschaft, an unserer JIA-Umfrage teilzunehmen. Wir möchten herausfinden, ob es Unterschiede in den **bevorzugten Behandlungsmethoden für die Juvenile Idiopathische Arthritis** in verschiedenen Ländern mit vergleichbaren Gesundheitssystemen und einer Subspezialisierung für Pädiatrische Rheumatologie gibt. Wir fragen Kinderrheumatologen in **Deutschland, Österreich, der Schweiz und Kanada**. Selbstverständlich werden die Ergebnisse anonym ausgewertet. Wir möchten hervorheben, dass es keine ‘richtigen’ Antworten gibt; alle genannten Alternativen entsprechen derzeit praktizierten Behandlungen.

Herzlichen Dank für Ihre Mitarbeit!

Dr. med. Boris Hügle
Dr. med. Susanne Benseler
The Hospital for Sick Children, Toronto, Kanada

Verwendete Abkürzungen: JIA – Juvenile Idiopatische Arthritis; NSAR – Nichtsteroidale Antirheumatika; DMARD – Disease-Modifying Anti-Rheumatic Drug (langwirksame Antirheumatika); ANA – Anti-nukleäre Antikörper; RF – Rheumafaktor; BSG - Blutsenkungsgeschwindigkeit

Bitte beantworten Sie die folgenden kurzen Fragen zu Ihrer Person.

1. Bitte geben Sie Ihre Email-Adresse an.

2. Bitte geben Sie Ihr derzeitiges Alter ein.

Alter in Jahren

3. Bitte geben Sie Ihr Geschlecht ein.

- weiblich
- männlich

4. Welche Ausbildung in Pädiatrischer Rheumatologie haben Sie erhalten?

- Arzt in Facharztweiterbildung
- Facharzt für Kinderheilkunde
- Zusatzbezeichnung “Kinder-Rheumatologe” oder vergleichbar

Andere (bitte angeben)

5. In welchem Jahr haben Sie Ihren Universitätsabschluss in Humanmedizin erhalten?

Jahr des Universtätsabschlusses

6. Bitte beschreiben Sie Ihr Tätigkeitsfeld:

- Universitätskrankenhaus
- Krankenhaus, nicht-universitär
- Selbständige Niederlassung

Andere (bitte angeben)

7. In welchem Land praktizieren Sie hauptsächlich?

- Deutschland
- Österreich
- Schweiz

Andere (bitte angeben)

A *Bitte betrachten Sie den folgenden hypothetischen Fall:* Ein **3 Jahre altes Mädchen** wird von ihren Eltern in Ihrer Praxis vorgestellt. Sie berichtet, dass sie seit zwei Monaten ein **geschwollenes rechtes Knie** hat und hinkt, insbesondere morgens. Die weitere Anamnese ergibt weder ein vorangegangenes Trauma noch eine vorangegangene Infektion. In der körperlichen Untersuchung finden Sie einen **Gelenkerguss in beiden Knien und beiden Knöchelgelenken** ohne Kontraktur, mit einer ansonsten normalen organ- und gelenkbezogenen Untersuchung. Die Laboruntersuchung zeigen ein normales Blutbild, Leber- und Nierenfunktionsparameter sowie Entzündungsparameter und einen **positiven ANA-Titer** mit negativem Rheumafaktor und negativem HLA-B27. Eine Augenuntersuchung (einschliesslich Spaltlampe) vor 2 Tagen war unauffällig. Sie stellen die Diagnose einer oligoartikulären JIA.

8. Welche der folgenden Medikamente würden Sie vorzugsweise in der Erstbehandlung verwenden?

- NSAR
- Intraartikuläre Gelenkinjektionen mit Kortikosteroiden
- DMARD
- Orale Kortikosteroide
- Biologika

Andere (bitte angeben)

9. Was würden Sie in Bezug auf Krankengymnastik und körperliche Aktivität bei diesem Patienten empfehlen?

- Regelmässige wöchentliche Physiotherapie durch einen ausgebildeten Physiotherapeuten (einschliesslich Übungen zu Hause)
- Kontinuierliche Übungen zu Hause nach anfänglicher Betreuung durch den Arzt oder einen ausgebildeten Physiotherapeuten
- Ermutigung zu vermehrter körperlicher Aktivität zu Hause

Andere (bitte angeben)

B *Bitte betrachten Sie den folgenden hypothetischen Fall:* Ein Patient mit derselben Vorgeschichte wie der unter A beschriebene Fall wird von einem anderen Pädiatrischen Rheumatologen mit einem **NSAR** behandelt. Sie wird nach einem Umzug der Familie **6 Monate nach Beginn ihrer Symptome** in Ihrer Praxis vorgestellt. Sie klagt noch immer über Morgensteifigkeit, und Sie finden **Gelenkergüsse in beiden Knien**. Die sonstige Untersuchung einschliesslich der Knöchel ist unauffällig. Eine kürzliche durchgeführte Augenuntersuchung war ebenfalls unauffällig.

10. Welche der folgenden Medikamente würden Sie vorzugsweise zu diesem Zeitpunkt verwenden?

- NSAID
- Intraartikuläre Gelenkinjektionen mit Kortikosteroiden
- DMARD
- Orale Kortikosteroide
- Biologika

Andere (bitte angeben)

C *Bitte betrachten Sie den folgenden hypothetischen Fall:* Ein Patient mit derselben Vorgeschichte wie der unter A beschriebene Fall wird von einem anderen Pädiatrischen Rheumatologen mit einem **NSAR** behandelt. Sie wird nach einem Umzug der Familie **6 Monate nach Beginn ihrer Symptome** in Ihrer Praxis vorgestellt. Sie hat **keine Gelenkbeschwerden** mehr, aber wurde 3 Monate nach Diagnosestellung mit einer **chronischen anterioren Uveitis** diagnostiziert, die **therapierefraktär auf topische Steroide** ist. Die organ- und skelettbezogene Untersuchung einschliesslich Knie und Knöchel ist unauffällig.

11. Welche der folgenden Medikamente würden Sie vorzugsweise zu diesem Zeitpunkt verwenden?

- NSAR
- DMARD
- Orale Kortikosteroide
- Biologika

Andere (bitte angeben)

Bevorzugte Behandlungsmethoden bei JIA in Deutschland und Kanada

D *Bitte betrachten Sie den folgenden hypothetischen Fall:* Ein **14 Jahre alter weiblicher Patient** stellt sich in Ihrer Praxis vor. Sie berichtet, dass sie in den letzten drei Monaten zunehmende Schwellungen beider Knie-, Knöchel-, Ellenbogen- und Handgelenke beobachtet hat, begleitet von Morgensteifigkeit für etwa 2 Stunden. Die weitere Anamnese ergibt weder ein vorangegangenes Trauma noch eine vorangegangene Infektion. In der körperlichen Untersuchung finden Sie **Gelenkergüsse in insgesamt 9 Gelenken**, mit einer ansonsten normalen organ- und gelenkbezogenen Untersuchung. Die Laboruntersuchung zeigen ein normales Blutbild, Leber- und Nierenfunktionsparameter, erhöhte Entzündungsparameter (BSG) und einen **positiven ANA-Titer mit negativem Rheumafaktor** und negative HLA-B27. Eine Augenuntersuchung (einschliesslich Spaltlampe) vor 2 Tagen war unauffällig. Sie stellen die Diagnose einer **seronegativen polyartikulären JIA**.

12. Welche der folgenden Medikamente würden Sie vorzugsweise in der Erstbehandlung verwenden?

- NSAR
- Intraartikuläre Gelenkinjektionen mit Kortikosteroiden
- DMARD
- Orale Kortikosteroide
- Biologika

Andere (bitte angeben)

13. Was würden Sie in Bezug auf Krankengymnastik und körperliche Aktivität bei diesem Patienten empfehlen?

- Regelmässige wöchentliche Physiotherapie durch einen ausgebildeten Physiotherapeuten (einschliesslich Übungen zu Hause)
- Kontinuierliche Übungen zu Hause nach anfänglicher Betreuung durch den Arzt oder einen ausgebildeten Physiotherapeuten
- Ermutigung zu vermehrter körperlicher Aktivität zu Hause

Andere (bitte angeben)

E *Bitte betrachten Sie den folgenden hypothetischen Fall:* Ein Patient mit derselben Vorgeschichte wie der unter D beschriebene Fall wird von einem anderen Pädiatrischen Rheumatologen mit DMARD behandelt. Sie wird nach einem Umzug der Familie in Ihrer Praxis vorgestellt, **einen Monat nach Behandlungsbeginn mit DMARD**. Sie hat noch immer Gelenkbeschwerden, und die körperliche Untersuchung zeigt **8 aktive Gelenke**.

14. Angenommen die unter E beschriebene Patientin zeigt auch in Folgeuntersuchungen einen unveränderten Befund, annähernd wie lange würden Sie nach Beginn einer Behandlung mit einem DMARD warten, bevor Sie Änderungen an der Behandlung vornehmen?

	1 Monat	2 Monate	3 Monate	4 Monate	6 Monate	9 Monate	12 Monate	18 Monate	24 Monate
Zeit									

15. Wie würden Sie zum Zeitpunkt, den Sie in der letzten Frage angegeben haben, Ihre Behandlung für die unter E beschriebene Patientin vorzugsweise ändern?

- Umstieg auf ein anderes DMARD-Präparat
- Behandlungsbeginn mit einem zusätzlichen DMARD-Präparat
- Behandlung mit intraartikulären Kortikosteroiden
- Umstieg auf oder zusätzliche Behandlung mit einem Biologikum

Andere (bitte angeben)

16. Angenommen die unter E beschriebene Patientin zeigt auch in Folgeuntersuchungen einen unveränderten Befund, annähernd wie lange würden Sie mit NSAID, DMARD oder Steroiden behandeln, bevor Sie eine Behandlung mit Biologika in Betracht ziehen?

	1 Monat	2 Monate	3 Monate	4 Monate	6 Monate	9 Monate	12 Monate	18 Monate	24 Monate
Zeit									

In den folgenden Fragen interessieren wir uns für die **von Ihnen bevorzugten Medikamente** innerhalb der entsprechenden Gruppen.

17. Gesetzt den Fall, Sie würden ein NSAR in der Behandlung eines 3 Jahre alten Mädchens mit oligoartikulärer JIA (ANA positiv, HLA-B27 negativ, RF negativ) ohne Uveitis einsetzen, welches wäre Ihr Medikament erster Wahl?

- Naproxen
- Indomethacin
- Ibuprofen
- Diclofenac
- Piroxicam

Andere (bitte angeben)

18. Gesetzt den Fall, Sie würden ein NSAR in der Behandlung einer 13 Jahre alten weiblichen Patienten mit polyartikulärer JIA (ANA positiv, HLA-B27 negativ, RF negativ) ohne Uveitis einsetzen, welches wäre Ihr Medikament erster Wahl?

- Naproxen
- Indomethacin
- Ibuprofen
- Diclofenac
- Piroxicam

Andere (bitte angeben)

19. Welches Kortikosteroid-Präparat würden Sie für eine intraartikuläre Injektion des Knies bei oligoartikulärer JIA bevorzugt einsetzen?

- Triamcinolonacetonid (z.B. Volon A®)
- Triamcinolonhexacetonid (z.B. Lederlon®)
- Betamethasone Acetate (z.B. Celeston-Depot®)
- Dexamethason (z.B. Lipotalon®)
- Methylprednisolone Acetate (z.B. Depo-Medrol®)

Andere (bitte angeben)

20. Gesetzt den Fall, Sie würden ein DMARD in der Behandlung einer 13 Jahre alten weiblichen Patienten mit polyartikulärer JIA (ANA positive, HLA-B27 negativ) ohne Uveitis einsetzen, welches wäre Ihr Medikament erster Wahl?

- Methotrexat
- Leflunomid
- Cyclosporin A
- Mycophenolat Mofetil oder EC-coated Mycophenolat
- Sulfasalazin

Andere (bitte angeben)

21. Gesetzt den Fall, Sie würden ein Biologikum in der Behandlung einer 13 Jahre alten weiblichen Patienten mit therapierefraktärer polyartikulärer JIA (ANA positive, HLA-B27 negativ, RF negativ) ohne Uveitis einsetzen, welches wäre Ihr Medikament erster Wahl?

- Infliximab (Remicade®)
- Etanercept (Enbrel®)
- Adalimumab (Humira®)
- Abatacept (Orencia®)

Andere (bitte angeben)

Herzlichen Dank für Ihre Mitarbeit in dieser Umfrage!
