# Supplementary material for: Treatment preferences in juvenile idiopathic arthritis – a comparative analysis in two health care systems
Source: Pediatr Rheumatol Online J. 2013 Jan 15;11:3. doi: 10.1186/1546-0096-11-3 (PMC3573942; doi:10.1186/1546-0096-11-3)
Supplement: Additional file 2 — Text of electronic survey questionnaire from SurveyMonkey, german. [file 1546-0096-11-3-S2.pdf]

# Approaches to treatment of seronegative JIA in Canada and Germany

Dear colleague,

thank you for participating in this survey. We want to examine any differences in **preferences in treatment for Juvenile Idiopathic Arthritis** in countries with comparable health systems and certified Subspecialists in Pediatric Rheumatology. We are surveying Pediatric Rheumatologists in **Canada and Germany, Austria & Switzerland**. Results will be analyzed anonymously. We would like to emphasize that there are no 'right' answers; all options presented are alternatives currently practised.

Thank you for your time!


*Boris Hugle*
*Susanne Benseler*
*The Hospital for Sick Children, Toronto*


Abbreviations used: JIA – Juvenile Idiopathic Arthritis; NSAID – Non-Steroidal Anti-Inflammatory Drug; DMARD – Disease-Modifying Anti-Rheumatic Drug; ANA – Anti-Nuclear Antibodies; RF – Rheumatoid Factor; ESR – Erythrocyte Sedimentation Rate

Please answer these brief questions about your person.

## 1. Please enter your email adress.

## 2. Please state your age.

Age in years

## 3. Please state your gender.

- Female
- Male

## 4. What is your level of training in Pediatric Rheumatology?

- Resident
- Specialty Degree in Pediatrics
- Subspecialty Degree in Pediatric Rheumatology

Other (please specify)

## 5. What year did you graduate from medical school?

Graduation year

### 6. Please describe your type of practice.

- Hospital-based, academic
- Hospital based, non-academic
- Community based

Other (please specify)

### 7. In which country do you mainly practice?

- Canada
- United States of America

Other (please specify)

A *Please consider the following hypothetical patient:* A **3 year old girl** is presented in your practice. The parents report that for the last 2 months she has had a **swollen right knee** and is limping, especially in the morning. Further history elicits no trauma or preceding infection. On examination you find an **effusion in both knees and both ankles with no contracture**, with an otherwise normal general and musculoskeletal examination. Bloodwork shows normal CBC, liver/kidney parameters and inflammatory parameters, and a **positive ANA**, but a negative RF and negative HLA-B27. An eye examination (including slit lamp examination) 2 days previously was unremarkable. You diagnose her with **oligoarticular JIA**.

### 8. Which of the following medications would you prefer for your initial treatment?

- NSAID
- Intraarticular corticosteroid joint injections
- DMARD
- Oral Corticosteroids
- Biologic treatment

Other (please specify)

### 9. What would be your recommendation regarding physiotherapy and activity in this patient?

- Regular weekly physiotherapy by a trained physiotherapy professional (with home exercise)
- Continued home exercise after initial coaching by the doctor or a trained physiotherapy professional
- Encourage a high level of physical activity at home

Other (please specify)

B *Please consider the following hypothetical patient:* A patient with the same characteristics as the one described under A was treated by another Pediatric Rheumatologist with an **NSAID**. After her family moved, she presents in your office, **6 months after onset of her symptoms**. She still complains of morning stiffness, and you find an **effusion in both knees**. The remaining examination including the ankles is unremarkable. A recent eye examination was unremarkable.

## 10. Which of the following medications would you prefer for treatment at this time ?

- NSAID
- Intraarticular corticosteroid joint injections
- DMARD
- Oral Corticosteroids
- Biologic treatment

Other (please specify)

C *Please consider the following hypothetical patient:* A patient with the same characteristics as the one described under A was treated by another Pediatric Rheumatologist with an **NSAID**. After her family moved, she presents in your office, **6 months after onset of her symptoms**. She has **no joint complaints**, but she was diagnosed with **chronic anterior uveitis refractory to topical steroids** 3 months after her initial visit. The musculoskeletal examination including ankles and knees is unremarkable.

## 11. Which of the following medications would you prefer for treatment at this time ?

- NSAID
- DMARD
- Oral Corticosteroids
- Biologic treatment

Other (please specify)

D *Please consider the following hypothetical patient:* A **14 year old female patient** presents in your office. She reports that for the last 3 months she has increasing swelling of both knees, ankles, wrists and elbows, with morning stiffness lasting for approximately 2 hours. Further history elicits no trauma or preceding infection. On examination you find **effusions in a total of 9 joints**, with an otherwise normal general and musculoskeletal examination. Bloodwork shows normal CBC, liver and kidney parameters, elevated inflammatory parameters (ESR), and a **positive ANA, but a negative RF** and negative HLA-B27. An eye examination (including slit lamp examination) 2 days previously was unremarkable. You diagnose her with **seronegative polyarticular JIA**.

**12. Which of the following medications would you prefer for initial treatment?**

- NSAID
- Intraarticular corticosteroid joint injections
- DMARD
- Oral Corticosteroids
- Biologic treatment

Other (please specify)

**13. What would be your recommendation regarding physiotherapy and activity in this patient?**

- Regular weekly physiotherapy by a trained physiotherapy professional (with home exercise)
- Continued home exercise after initial coaching by the doctor or a trained physiotherapy professional
- Encourage a high level of physical activity at home

Other (please specify)

E *Please consider the following hypothetical patient:* A patient with the same characteristics as the one described in D was treated by another Pediatric Rheumatologist with a **DMARD**. After her family moved, she presents in your office, **one month after start of DMARD treatment**. She has ongoing joint complaints, and the physical examination still shows **8 active joints**.

**14. Assuming that the patient described in E remains unchanged on subsequent presentations, approximately how long after institution of the DMARD treatment would you wait before you would consider changing her management?**

| | 1 month | 2 months | 3 months | 4 months | 6 months | 9 months | 12 months | 18 months | 24 months |
|---|---|---|---|---|---|---|---|---|---|
| Time | | | | | | | | | |

**15. At the time point you gave in the last question, what would be your preferred choice for changing the management of the patient described in E?**

- Switch to a different DMARD
- Treat with an additional DMARD
- Treat with intraarticular Steroids
- Add or switch to a biologic agent

Other (please specify)

**16. Assuming that the patient described in E remains unchanged on subsequent presentations, approximately how long would you treat with NSAID, DMARD or steroids before you would consider a biologic agent?**

| | 1 month | 2 months | 3 months | 4 months | 6 months | 9 months | 12 months | 18 months | 24 months |
|---|---|---|---|---|---|---|---|---|---|
| Time | | | | | | | | | |

The following questions are designed to assess **your preferences for various medications** within their respective groups.

**17. If you would use an NSAID for treatment of a 3 year old female patient with oligoarticular JIA (ANA positive, HLA-B27 negative, RF negative) with no uveitis, which would be your initial choice?**

- Naproxen
- Indomethacin
- Ibuprofen
- Diclofenac
- Piroxicam

Other (please specify)

**18. If you would use an NSAID for treatment of a 13 year old female patient with polyarticular JIA (ANA positive, HLA-B27 negative, RF negative) with no uveitis, which would be your initial choice?**

- Naproxen
- Indomethacin
- Ibuprofen
- Diclofenac
- Piroxicam

Other (please specify)

**19. Which would be the corticosteroid preparation of your choice for intraarticular steroid injection of the knee in oligoarticular JIA?**

- Triamcinolone Acetonid (e.g. Kenalog®)
- Triamcinolone Hexacetonide (e.g. Aristospan®)
- Betamethasone Acetate (e.g. Soluspan®)
- Dexamethasone (e.g. Decadron®)
- Methylprednisolone Acetate (e.g. Depo-Medrol®)

Other (please specify)

**20. If you would use a DMARD for treatment of a 13 year old female patient with polyarticular Juvenile Idiopathic Arthritis (ANA positive, HLA-B27 negative, Rheumatoid Factor negative) with no uveitis, which would be your initial choice?**

- Methotrexate
- Leflunomide
- Cyclosporine
- Mycophenolate Mofetil or enteric-coated Mycophenolate
- Sulfasalazine

Other (please specify)

**21. If you would use a biologic agent for treatment of a 13 year old female patient with refractory polyarticular JIA (ANA positive, HLA-B27 negative, rheumatoid factor negative) with no uveitis, which would be your initial choice?**

- Infliximab (Remicade®)
- Etanercept (Enbrel®)
- Adalimumab (Humira®)
- Abatacept (Orencia®)

Other (please specify)

Thank you for taking the time to complete this survey!
